# Synthesis of Polydopamine Hollow Capsules via a Polydopamine Mediated Silica Water Dissolution Process and Its Application for Enzyme Encapsulation

**DOI:** 10.3389/fchem.2019.00468

**Published:** 2019-07-03

**Authors:** Huy Quang Tran, Mrinal Bhave, Guowang Xu, Chenghua Sun, Aimin Yu

**Affiliations:** Department of Chemistry and Biotechnology, Faculty of Science, Engineering and Technology, Swinburne University of Technology, Hawthorn, VIC, Australia

**Keywords:** polydopamine, hollow capsules, mesoporous silica, green synthesis, enzyme encapsulation

## Abstract

Herein, we present a systematic study on the preparation of polydopamine (PDA) hollow capsules by templating silica particles which were subsequently removed by a PDA mediated water dissolution process without using any harsh chemical treatment. It was found that the time required for silica removal varied depending on the PDA coating and dissolution conditions. Factors that could influence the core removal process including the PDA thickness and coating temperature, silica calcination duration and the availability of water were then examined in detail. Additionally, catalase was used as a model enzyme to be encapsulated into PDA hollow capsules and its bio-functionality was found to remain active. The bioactivity test results also indicated that the as-synthesized PDA capsules possessed a porous structure, which allows the penetration of small molecules such as H_2_O_2_. This study offers a better insight into silica dissolution process that mediated by PDA and contributes to the development of an eco-friendly approach for the fabrication of hollow capsules that have promising applications in drug delivery systems.

## Introduction

The fabrication and utilization of polymeric hollow capsules with high drug loading capacity and a controllable drug release profile have received enormous attention in the field of controlled drug delivery systems (Kumar et al., 2007; Hoffman, 2008; Mura et al., 2013). Multiple attempts have been devoted to the development of diverse triggered-release systems using different polymeric components; however, some challenges still remain, such as the loading capacity and functionality of loaded molecules.

Polydopamine (PDA), a polymeric form of dopamine (DA) monomer, has been utilized as a promising polymeric material for various purposes. For example, the utilization of PDA coating on polyethylene to enhance the power capacities of batteries was previously mentioned by Ryou et al. (2011) or the incorporation of PDA nanoparticles within PEGylated borate-coordination-polymer system for cancer therapy was described by Liu et al. (2018). In the context of drug delivery purposes, the employment of PDA coating onto sacrificial templates for the fabrication of hollow capsules has many benefits; for instance, it can adhere onto many types of particulate particles and renders the ability to control the thickness of obtained capsules through its polymerization process, and it was previously proven for its excellent biocompatibility (Chen et al., 2014; Ryu et al., 2018; Wu et al., 2018). In addition, the surface chemistry of PDA contains multiple amine groups, which can facilitate further surface modifications to enhance the selectivity of synthesized capsules (Chang et al., 2016; Wang et al., 2016; Wei et al., 2017).

Despite the convenience and benefits of PDA coating, its use to fabricate hollow capsules still has some drawbacks, which mainly arise from the sacrificial core removal process due to the requirement of harsh chemical treatment (Cui et al., 2015; Hong et al., 2017; Zhang et al., 2017). For example, silica particle is a commonly used template for making hollow capsules and hydrofluoric acid (HF) put in long form at first use is required to completely dissolve the silica. Recently, a novel approach using PDA coating to aid the silica particles dissolution in water was reported by Nador et al. (2017). It was suggested that polyamines and/or polyimines originating from the polymerization of DA monomer could assist the formation of selective catechol–silica [Si(C_6_H_4_O_2_)_3_], which promotie the dissolution rate of silica up to 15-25 folds (Mizutani et al., 1993; Patwardhan et al., 2011; Nador et al., 2017). This finding is very beneficial for the development of drug delivery system in term of enhancement of drug loading capacity as mesoporous silica nanoparticles (MSN) is well-known for its high drug loading capacity (Yu et al., 2005; Plush et al., 2009), and its subsequent disintegration in water after PDA coating would significantly reduce the effect on the fundamental functionality of encapsulated molecules.

So far, there is still little research on the study of the potential parameters that might influence the silica dissolution process. With this in mind, a systematic study to evaluate the effects of various parameters on silica dissolution process mediated by PDA and water is conducted. Specifically, the effect of factors that involve PDA coatings, such as PDA coating thickness and temperature, and the rigidity of the sacrificial silica core template was chosen to study. In addition, the influence of water requirement for silica removal process was also of interest.

The remaining integrity and functionality of the encapsulated molecules within hollow capsules are always important for the development of a drug delivery system. In this context, catalase was chosen as a model biomacromolecule to be encapsulated into PDA capsules and its bio-functionality was evaluated to study whether the silica core removal has any effect on the bioactivity of encapsulated enzyme.

## Experimental Section

### Reagents and Materials

Dopamine hydrochloride (DA), ammonia hydroxide (NH_4_OH, 28–30%), ethanol (EtOH), 2-propanol (iPrOH), tetraethyl orthosilicate (TEOS), cetyltrimethylammonium bromide (CTAB), Pluronic F127, 3-(aminopropyl) triethoxysilane (APTES), catalase (C30), fluorescein isothiocyanate (FITC), hydrogen peroxide (H_2_O_2_), dimethyl sulfoxide (DMSO), and sodium azide (NaN_3_) were purchased from Sigma-Aldrich Australia. Phosphate buffer (PB) solutions with different pH were made by mixing 0.2 M NaH_2_PO_4_ with Na_2_HPO_4_ and adjusted to appropriate pH using 2.0 M HCl and 2.0 M NaOH solutions. Milli-Q water (18.2 MΩ cm) was utilized in all the experiments.

### Synthesis of Mesoporous Silica Nanoparticles (MSN)

MSN particles were prepared by a sol-gel process with binary surfactant system previously described by Kim et al. (2010). In brief, 0.5 g of CTAB and 2.05 g of Pluronic F127 were dissolved in a mixture of water (96 mL), EtOH (43.1 mL), and concentrated NH_4_OH solution (28 wt. %) (11.2 mL) by stirring at room temperature (RT). After obtaining a well-dispersed homogenous solution, 1.93 mL of TEOS was added quickly into the mixture under vigorous stirring for 90 s. The mixture was kept static for the next 24 h at RT for further condensation. The white precipitate was then collected, washed three times with water, and calcined at 550°C for different time intervals (2, 4, and 6 h) to remove the surfactants.

### Surface Modification of MSN by Amine Functional Groups

The functionalization of bare MSN with amine groups followed previous work described by Huang et al. (2003) with slight modifications. Briefly, 0.2 g of MSN was refluxed with 10 mL of toluene and 1 mL of APTES under stirring at 70°C for 18 h. The white precipitate was then collected via centrifugation and washed with toluene and pentane, respectively.

### Coating MSN With PDA

A previous procedure of PDA coating (Nador et al., 2017) was followed with slight modifications. Briefly, 57 mg DA was dissolved in 39 mL iPrOH. Then, 2.25 mL NH_4_OH and 30 mg MSN were added to the solution. The coating process was left for 18 or 24 h under magnetic stirring at RT. The MSN@PDA particles were obtained by centrifugation and washed several times with EtOH.

### Dissolution of the Silica Core of MSN@PDA in Water

For MSN core removal, MSN@PDA particles were dispersed in different water volumes. The vial was kept under stirring for 48 h in an incubator with set temperature at 37°C. The PDA hollow capsules were obtained via centrifugation and washed 3 times by water.

### Preparation of FITC-Labeled Catalase

The conjugation of FITC and catalase followed the previous procedure described by Yu et al. (2005) with slight modifications. In brief, 300 μl of FITC/DMSO (1 mg/mL) was added to 4 mL of 1 mg/mL catalase solution in PB (pH 8.0). The mixture was allowed to stand for 50 min, followed by the dialysis against PB (pH 7.0) for 72 h, changing the solution every 24 h.

### Encapsulation of FITC-Catalase in PDA Hollow Capsules

Approximately 4 mg of amine-grafted MSN particles were added and incubated with 4 mL of 1 mg/mL FITC-catalase solution at room temperature for 24 h. The FITC-catalase loaded MSN were then washed with PB (pH 7.4) to remove unbound enzyme. These particles were later subjected to PDA coating at RT and core removal process as described above.

### Evaluation of Enzyme Activity After Encapsulation

An amount of 50 μL of free PDA capsule and catalase@PDA capsule solution (5 mg/mL) was added to a solution of 10 mM H_2_O_2_ in PB pH 7. The activity of catalase was examined based on the absorbance change at 240 nm of the H_2_O_2_ solution, in response to the addition of free PDA capsules and catalase@PDA capsules.

### Material Characterization

The UV-Visible spectroscopy was carried out with a Cary 5000 UV-Vis spectrophotometer (Australia). FTIR was performed on MSN and PDA@MSN to check the silanol bonds in the silica network and success of PDA coating. It was performed using a Thermo Scientific Nicolet iS5 FT-IR Spectrometer, with 8 scans being performed per test.

Morphology of MSN and PDA hollow capsules were examined by using a field emission scanning electron microscope (FeSEM, ZEISS SUPRA 40VP, Germany) at an acceleration voltage of 3 kV.

Additionally, transmission electron microscopy (TEM) and energy dispersive X-ray spectroscopy (EDS) was utilized to observe of the interior structure of MSN@PDA after being dispersed in water as well as to quantify the remaining Si element. TEM and EDS were performed by using a JEM 2100F (JEOL Ltd., Japan) at room temperature at a voltage of 200 kV.

To confirm the successful loading of FITC-catalase into PDA capsules, visualization of FITC fluorescence were performed using Olympus Fluoview 1000 IX81 (Japan) confocal laser scanning microscope, operated using 100× oil-immersion objective combined with 5× optical zoom.

## Results and Discussion

### PDA Coating of Mesoporous Silica Nanoparticles (MSN)

**Figure 1:**
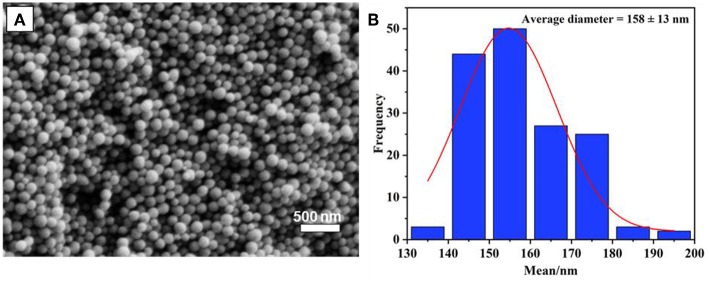
**(A)** SEM image of synthesized MSN nanoparticles, and size distribution **(B)**.

The preparation of monodispersed MSN was reproduced by a sol-gel process using a binary surfactant system with the aid of triblock copolymer Pluronic F127 and CTAB described by Kim et al. (2010). In this process, TEOS plays as the main source of silica precursor. As could be seen in Figure 1, the obtained particles were quite uniform, with a spherical shape, and their size distribution was calculated to be 158 ± 13 nm based on an average of 100 particles. It was found that the calcination time at 550°C (2, 4, or 6 h) does not significantly affect the size of synthesized MSN. However, as per a previous report, calcination time might affect the surface area and rigidity of particles due to the condensation of the Si-O-Si network (Mokaya, 1999; Ruckdeschel et al., 2015).

**Figure 2:**
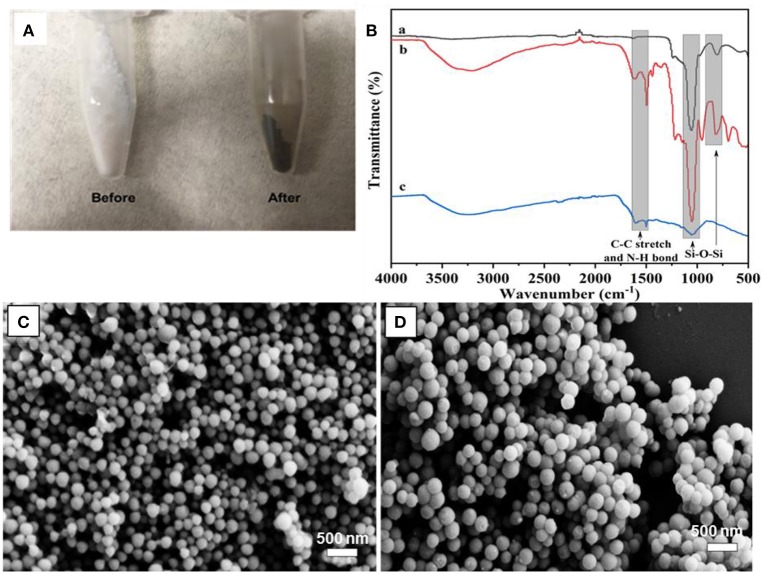
**(A)** Photo images of MSN before and after PDA coating, **(B)** FT-IR spectra of MSN (a), MSN@PDA (b) and MSN@PDA after 48 h water dispersion (c), and SEM image of **(C)** MSN@PDA particles after 18 h-PDA coating at 25°C and **(D)** MSN@PDA particles after 24 h-PDA coating at 45°C.

The PDA coating on MSN took place in a DA solution under mild basic conditions, with the assistance of oxygen. It has been suggested that the formation of PDA has consecutive steps, initiated by the oxidization of DA monomer, followed by further oxidization, cyclization, and rearrangement to form 5, 6-dihydroxyindole (Hong et al., 2012). The obtained 5, 6-dihydroxyindole products are then adsorbed onto MSN particles via covalent oxidative polymerization and non-covalent interactions, such as II-II interactions, electrostatic interactions and hydrogen bonding (Hong et al., 2012; García et al., 2014). The visual color change of MSN from white to dark-brown gives an indication of the successful coating of PDA (Figure 2A). To support for this, the FT-IR spectrum of MSN before coating shows two peaks in the range of 963–1,054 cm^−1^, which is attributed to the silanol bonding (Si-O-Si) in the silica network. After PDA coating, two additional peaks are observed in the range of 1,495–1,609 cm^−1^ due to the C-C stretching of benzene rings and the N-H bending of PDA (Figure 2B).

It was observed that the average size of MSN significantly increased after PDA coating, and the coating thickness was strongly affected by the coating conditions such as polymerization time. For example, the average size of MSN increased from 158 to 178 and 226 nm after being coated with PDA for 18 and 24 h, respectively. This equals to PDA coating thickness of 20 nm (18 h) and 68 nm (24 h). The PDA coating thickness was also affected by the coating temperature. For example, for the polymerization carried out at 45°C, the average sizes of PDA particles were 221 and 232 nm for 18 and 24 h coating condition, respectively. It could be reasoned that the deposition rate and the polymerization degree of the PDA layer were influenced by the coating temperature and the duration of the coating process (Jiang et al., 2011; Cho and Kim, 2015). Compared to the coating condition at ambient temperature, the polymerization of DA occurs at a higher rate, which results in thicker PDA coating. Of note, there was no aggregation of PDA-coated MSN regardless of different coating conditions, as shown in Figures 2C,D.

### Preparation of PDA Capsules by Dissolution of Silica Core

PDA hollow capsules were obtained by the removal of silica core templates by simply dispersing the MSN@PDA particles in water. However, we found that the silica dissipation process was strongly influenced by a few factors including dissolution conditions and the properties of MSN@PDA particle itself, such as the thickness of PDA coating layer and/or the rigidity of the interior core of MSN; see below for detailed studies.

#### Effect of PDA Coating Thickness on Silica Dissolution

**Figure 3:**
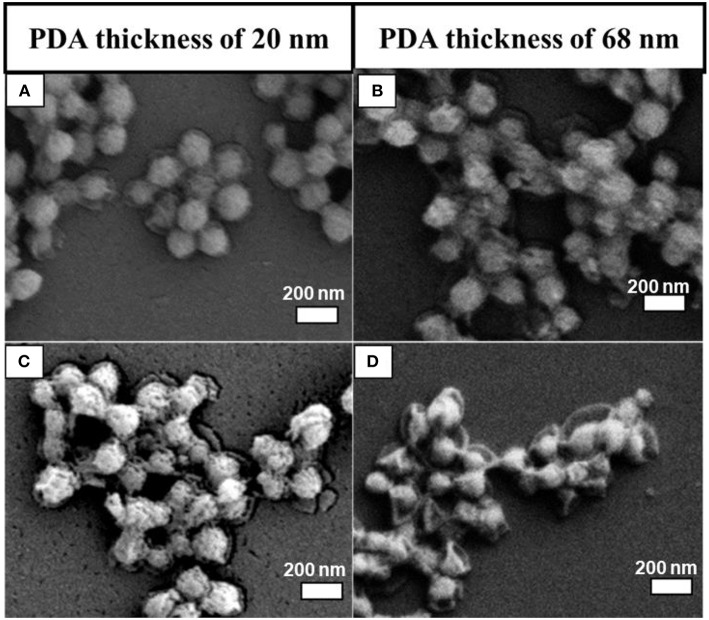
SEM images of MSN@PDA coated at room temperature with different PDA coating thickness after 24 h **(A,B)** and 48 h water dispersion **(C,D)**.

The PDA thickness effect was examined by dispersing two batches of as-prepared MSN@PDA particles with a thickness of 20 nm (coated for 18 h at RT) and 68 nm (coated for 24 h at RT) into the water for 1 and 2 days. The morphology change of MSN@PDA particles is shown in Figure 3. It was interesting to observe that the dissolution rate of silica increases with thicker coating; i.e., the silica core of PDA@MSN particles with 68 nm coating (D) dissolved more quickly than particles with 20 nm PDA coating (C). The reason might be that thicker PDA coating possesses more polyamine (or polyimine) groups on the particle, leading to the acceleration of the silica dissipation rate.

#### Effect of PDA Coating Temperature on Silica Dissolution

**Figure 4:**
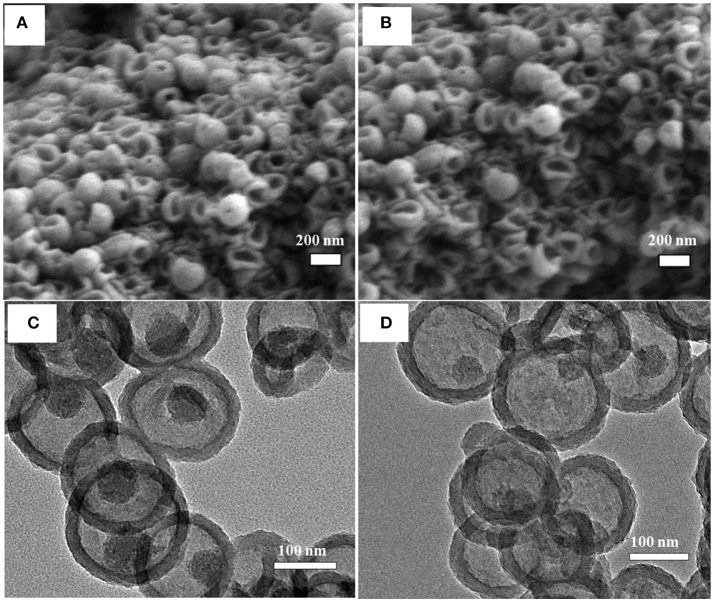
SEM **(A,B)** and TEM **(C,D)** images of MSN@PDA with PDA thickness of 74 nm that coated at 45°C after dispersed in water for 24 h **(A,C)** and 48 h **(B,D)**.

It has been indicated above that increasing the PDA coating temperature resulted in thicker PDA coating. Additionally, higher coating temperature might also affect the resulting structure of PDA, such as a higher polymerization degree and a more rigid structure. This was evidenced by the silica dissipation rate and morphology. Figure 4 shows MSN@PDA particles with 74 nm PDA coating (coated at 45°C) after dispersion in water for 24 and 48 h. Compared with MSN@PDA particles with similar PDA thickness (68 nm) but coated at RT (Figures 3B,D), the disintegration of silica core is much quicker. The removal of silica for PDA coating at RT could only be visible after 48 h. However, this phenomenon could be well-observed within 24 h when PDA was coated at 45°C. Of note, MSN@PDA particles coated at 45°C after core removal yielded hollow capsules with a more free-standing structure, while MSN@PDA coated at RT yielded collapsed capsules. This could be attributed to the higher polymerization degree, higher intensity and/or uneven deposition of PDA layer at 45°C. The disintegration of the interior MSN core was then confirmed under TEM analysis, which was displayed in Figures 4C,D. As could be seen, compared to the size of bare MSN, the silica core was dissolved up to 58% after the first 24 h water dispersion, and it continued to be removed up to 70% for the next 24 h. Of note, there was a discrepancy in the thickness of the PDA coating before and after the water dispersion process. The thickness of the PDA wall after being dispersed was calculated to be approximately 22 nm, which was much thinner than 74 nm of those before being dispersed. This discrepancy is likely due to the involvement of PDA wall to form complexes of selective catechol–silica [Si(C_6_H_4_O_2_)_3_] during the dissolution process.

#### Effect of MSN Calcination Time on Silica Dissolution

**Figure 5:**
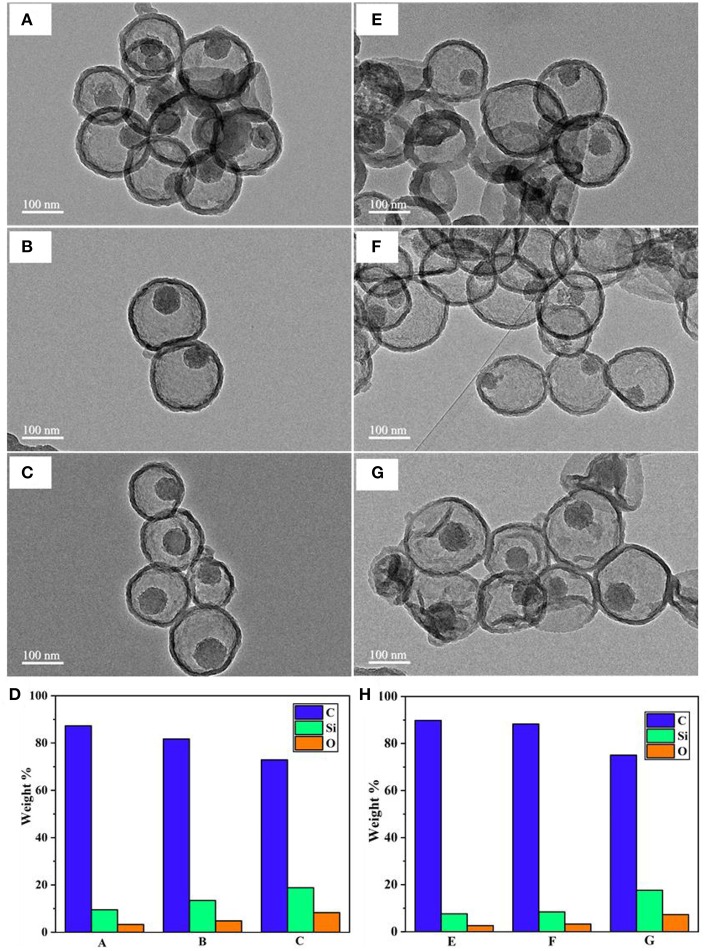
TEM images of MSN@PDA with different MSN calcination time: 2, 4, and 6 h after 24 h- (**A–C**, respectively) and 48 h-water dispersion (**E–G**, respectively), and their corresponding EDS analysis (**D,H**, respectively).

Another factor we found that influences the water disintegration of silica core is the polymerization and condensation degree of silanol bonding in the silica network. Previous studies suggested that the silica network synthesized by the Stober method contains various silica species, and most of them are Q3 and Q4, which represent trimers and tetramers (Zhang et al., 2010; Lunevich et al., 2016). The content of Q3/Q4 varies in regards to the calcination conditions during the surfactant removal process. With this in mind, an experiment was designed to investigate whether, at the same temperature conditions, different calcination durations have any effect on the silica removal process after coating. Under the same PDA coating conditions and water availability, MSN@PDA particles with the sacrificial silica calcined for 2 h exhibited a faster core dissolution compared to those of 4 and 6 h calcined silica cores (Figures 5A–H). Silica core was not observed in some capsules after 48 h water dispersion. This could be explained by the fact that the ratio of Q4 to Q3 increases in relation to the length of the calcination duration; specifically, the longer the calcination duration was, the more rigidity of the silica network (Ruckdeschel et al., 2015). To support this observation, the elemental analysis was carried out by using EDS. As expected, the content of Si element in the sample with the sacrificial core of 2 h-calcined MSN had the lowest weight percentage with 3.24 and 2.61% after 24 and 48 h water dispersion, respectively, whereas those figure of 6 h-calcined MSN samples were only 8.29 and 7.29%, respectively (Figures 5D,H). From the obtained results, it can be confirmed that a reduction in silica calcination can facilitate silica disintegration later on.

#### Effect of Water Amount on Silica Dissolution

**Figure 6:**
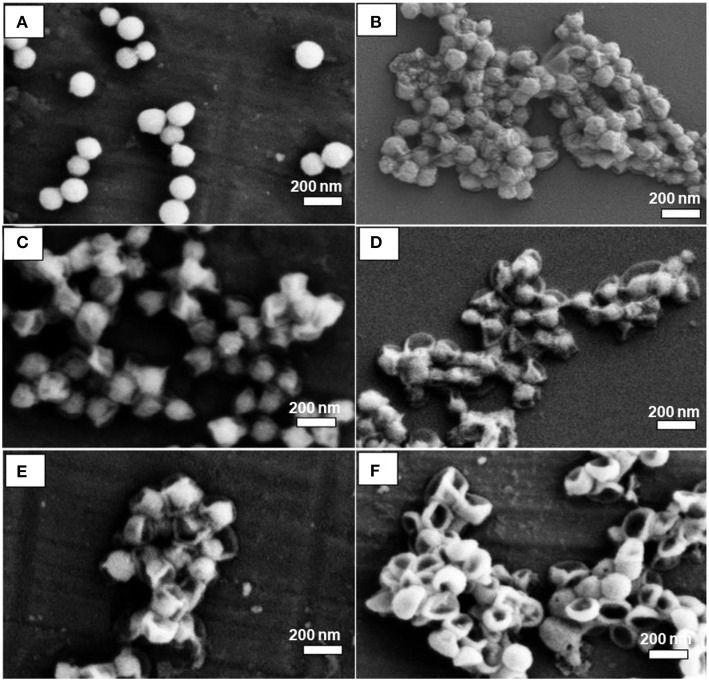
SEM images of MSN@PDA with PDA thickness of 68 nm after being dispersed in water at three different ratios: 1:1 **(A,B)**, 1:5 **(C,D)**, and 1:10 **(E,F)** after 24 h **(A,C,D)**, and 48 h **(B,D,F)**, respectively.

The silica dissipation was also affected by water availability during the removal process. To test this point of view, MSN@PDA particles with 68 nm PDA coating were dispersed in water for 2 consecutive days with three different ratios (1:1, 1:5, and 1:10 (MSN@PDA/H_2_O, mg/mL). As can be seen in Figure 6, the volume of water was a crucial factor for the silica core removal. At the ratio of 1:1, there was a slight dissolution of MSN, leaving the wrinkle of the PDA wall. The rate of dissolution significantly increased when more water was used. At the ratio of 1:10, the silica core was almost completely dissolved and hard to be seen under SEM.

To support this observation, the MSN@PDA after 48 h water dispersion were examined by FT-IR. The spectra revealed that the intensity of silica peak of MSN@PDA decreased significantly in the range around 1,054 cm^−1^, which represented the presence of silanol bonding (Si-O-Si) in silica network (Figure 2B). This also meant that the silica core could be largely dissolved. According to Igarashi et al. (2003), the regularity of loss of silica core involves adsorption of water onto the silanol groups located on the silica surface, which initiates the cleavage of the nearby Si–O–Si bonding, eventually resulting in the structural collapse and dissipation. In terms of silica structure, it is made of a condensed network of silanol bonding; hence, in order to speed up the cleaving process, water availability seems to be essential.

### Encapsulation of Enzyme in PDA Hollow Capsules

**Figure 7:**
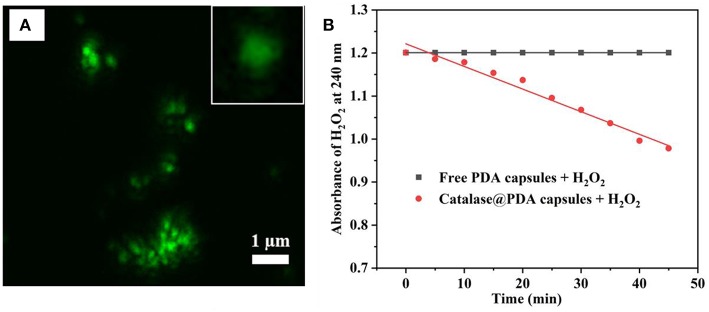
CLSM image of FITC-catalase@PDA capsules and single FITC-catalase@PDA capsules (inset) **(A)**, and catalytic activity of catalase toward 10 mM H_2_O_2_ **(B)**.

PDA hollow capsules were then assessed for their capacity to encapsulate macro-biomolecules. For this purpose, catalase was chosen as a model enzyme to be encapsulated into PDA hollow capsules using the pre-loading strategy that we developed previously (Yu et al., 2005). The encapsulation was carried out by first loading catalase onto MSN, followed by PDA coating and silica dissolution in water. Before catalase adsorption, MSN was grafted with amino groups in order to enhance the electrostatic interaction with catalase. The amount of catalase adsorbed onto amine-functionalized MSN was measured the UV-Vis absorbance of catalase before and after loading. It was determined that the amount of catalase adsorbed onto MSN was approximately 302 ± 78 mg/g of MSN. This amount was significantly higher than that for bare MSN (~75 mg/g). The catalase loaded MSN was subjected to PDA coating and silica core removal. The successful loading of catalase into PDA capsules was confirmed by the uniform fluorescence within the hollow capsule under CLSM when FITC-catalase was used (Figure 7A). It was previously reported that the illumination of protein with a fluorescent tag could be quenched by PDA (~80% quenching effectiveness) (Qiang et al., 2015; Xing et al., 2018); however, the fluorescence of FITC-catalase entrapped within PDA capsules could be observed to be quite vibrant. This is likely due to the high concentration of FITC-labeled protein being encapsulated.

One drawback of previous work on using silica nanoparticles for hollow capsule preparation is that harsh conditions such HF are needed to dissolve silica templates, which might affect the bioactivity of biomacromolecules. The advantage of the present work is that we are able to dissolve the silica core using water. Whether the encapsulated catalase retains its activity was examined by its capacity to interact with and decompose H_2_O_2._ This was carried out by measuring the absorbance of H_2_O_2_ at 240 nm upon the addition of catalase@PDA capsule. Empty PDA capsules at the same concentration was used as a control. As can be seen in Figure 7B, the catalytic activity of encapsulated catalase was prominent upon the addition to H_2_O_2_ solution, and the rate of absorbance change of H_2_O_2_ solution was calculated to be 0.006 per minute. This result suggests that the catalytic activity of entrapped catalase is still retained after the encapsulation and silica removal process, even though the breakdown of H_2_O_2_ occurred slowly and gradually since the interaction between encapsulated catalase and H_2_O_2_ molecules was reduced by the PDA layer. From the obtained results, it suggested that the PDA capsule wall was likely porous, which served as a pathway for H_2_O_2_ molecule penetration.

## Conclusion

We have demonstrated the successful preparation of PDA hollow capsules by templating mesoporous silica particles. After PDA coating, silica cores were able to be removed simply in water. The factors that influenced the rate of silica dissolution were studied in detail. It was found that the length of time required for silica removal process varied depending on the PDA coating temperature and duration. In addition, the amount of available water was also a factor attributing to the rate of silica disassembly. More importantly, the encapsulated catalase within PDA hollow capsules still retained its function to decompose its substrate H_2_O_2_. The outcomes of this study offer significant insights into silica dissolution process mediated by PDA and contributes to the development of an eco-friendly approach for hollow capsules fabrication which could be utilized as drug delivery systems to encapsulate various biomolecules and drugs, with retained bioactivity.

## Data Availability

The raw data supporting the conclusions of this manuscript will be made available by the authors, without undue reservation, to any qualified researcher.

## Author Contributions

HT conducted the synthesis experiment and wrote the draft paper. GX conducted the sample characterization. MB, CS, and AY were involved in the project design and paper writing.

### Conflict of Interest Statement

The authors declare that the research was conducted in the absence of any commercial or financial relationships that could be construed as a potential conflict of interest.
